# Long non-coding RNA PTENP1 interacts with miR-193a-3p to suppress cell migration and invasion through the PTEN pathway in hepatocellular carcinoma

**DOI:** 10.18632/oncotarget.22305

**Published:** 2017-11-06

**Authors:** Yu-Yuan Qian, Kun Li, Quan-Yan Liu, Zhi-Su Liu

**Affiliations:** ^1^ Department of Hepatobiliary and Pancreatic Surgery, Zhongnan Hospital of Wuhan University, Wuhan 430071, China

**Keywords:** hepatocellular carcinoma, PTENP1, miR-193a-3p, PTEN, ceRNA

## Abstract

Long non-coding RNA PTENP1, the pseudogene of PTEN tumor suppressor, was previously reported to be a tumour suppressor in some cancer types. However, the precise effects mediated by PTENP1 transcripts within intricate regulatory networks involving molecular interactions with PTEN and tumorigenicity in hepatocellular carcinoma (HCC) remains elusive. Here, we identify the critical biological functions of PTENP1 and discuss whether PTENP1 could directly interact with miR-193a-3p to affect the progression of HCC both *in vitro* and *in vivo*. We demonstrated that PTENP1 level in the HCC tissues was significantly lower compared with those in the adjacent normal tissues. And PTENP1 was able to repress cell invasion, metastasis, and proliferation capacity in HCC cell lines. The overexpression of PTENP1 inhibited HCC growth both *in vitro* and *in vivo*. There were a binding sequence and direct interaction between PTENP1 and miR-193a-3p. PTENP1 as an endogenous sponge interacted with miR-193a-3p, leading to regulate the downstream PTEN/Akt pathway.

These results suggested that PTENP1 with its suppression effect might serve as novel biomarkers and potent therapeutic strategies in HCC.

## INTRODUCTION

Hepatocellular carcinoma (HCC) has a poor prognosis and a high recurrence rate and is one of the most common malignant tumors in the world [1]. Various transcript factors and epigenetic changes are related to the invasion, proliferation and metastasis of HCC cells [2–4]. Therefore, in the search to decipher the molecular mechanisms of HCC cells that initiate and promote unrestricted tumor growth, especially the genetic and epigenetic changes, the carcinogenic character of microRNAs (miRNAs) and long non-coding RNA (lncRNAs) have attracted much attention in recent studies [5, 6].

Long noncoding RNAs (lncRNAs) act as non-protein-coding genes, are classified by a length of >200 nucleotides, and are related to multiple biological functions [7, 8]. In addition, the dysregulation of lncRNAs is related to vigorous tumor growth [8, 9].

As a suppressor of liver cancer, PTENP1 participates in repressing cell proliferation, inhibiting migration, and promoting apoptosis [10, 11]. Recent studies have shown that lncRNAs, as competing endogenous RNAs (ceRNA), play important roles in modulating miRNA function through binding sites [8, 12]. LncRNAs may interact with miRNAs by specifically competing with ceRNAs, leading to dysfunction in a variety of cancers [5, 13]. Similarly, we have found that PTENP1 influences the biological function of miR-193a-3p.

MiRNAs, such as miR-193a-3p, are small non-coding RNAs with a length of 20 nucleotides and regulate cell invasion, migration, and apoptosis [14]. It has been reported that repression of miR-193a-3p can increase PTEN expression, leading to the suppression of tumorigenesis and avoidance of metabolic disorders in malignant tumors [14].

Compared to adjacent normal liver tissues, our results show that the expression of PTENP1 is low and that the expression of miR-193a-3p is high in HCC tissues. In HCC, miR-193a-3p is a direct target of PTENP1, acting as a tumor suppressor by modulating downstream targets such as the PTEN/Akt signaling pathway.

In our study, we reveal that PTENP1 and miR-193a-3p have the potential to be new biomarkers and therapeutic targets for successful intervention in HCC. In addition, the biological function and mechanism of PTENP1 are still unclear and require more research.

## RESULTS

### PTENP1 has a low expression level in HCC tissues

**Figure 1:**
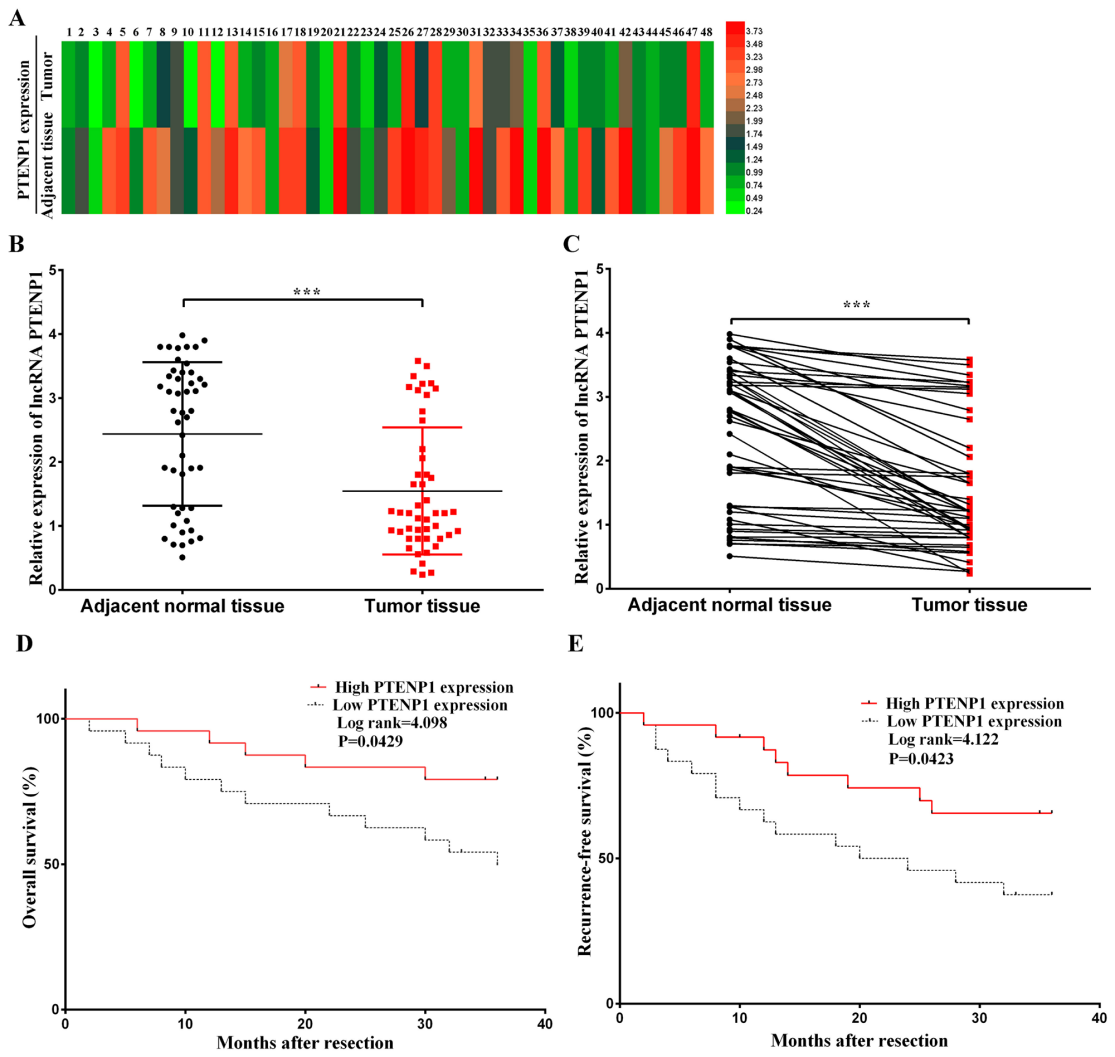
PTENP1 related to prognosis of patients after operation was broadly distributed in adjacent normal tissues at a relatively high level, while low-expressed on HCC cells **(A)** PTENP1 level was evaluated by qRT–PCR in 48 adjacent normal tissues and HCC samples. The expression level was normalized to that of GAPDH. PTENP1 was lowexpressed in HCC samples (P < 0.001). **(B)** and **(C)** Relative PTENP1 level was lower in HCC tissues compared with the paired adjacent normal tissues. The results were presented as mean ± SD from three independent experiments (^***^P < 0.001). **(D)** Overall survival and **(E)** recurrence-free survival were investigated and analyzed by Kaplan-Meier analysis from the 48 patients. And the high PTENP1 level group and low level group were divided and cut off at the median level of PTENP1 (^*^P< 0.05).

PTENP1 had a low expression level in HCC tissues compared with the adjacent normal tissues. In our study, the expression level of lncRNA PTENP1 was detected by qPCR in 48 paired tissues. The PTENP1 expression level was clearly low in tumorous tissues (P < 0.001; Figure 1A, 1B and 1C). Moreover, we discovered that the PTENP1 expression level was notably related to tumor size (*P* < 0.05) and TNM (tumor-regional lymph node-metastasis) stage (*P* < 0.05) in HCC patients. However, the PTENP1 expression level was not significantly associated with other clinicopathological characteristics such as age, gender, liver cirrhosis, PVST, and serum AFP (*P* > 0.05, Table 1). Furthermore, there was a longer overall survival (*P* < 0.05; Figure 1D) and substantially lower recurrence rate (*P* < 0.05; Figure 1E) in HCC patients with a high expression level of PTENP1 compared with patients with a low expression level of PTENP1. These results revealed that the high expression level of PTENP1 may be related to tumor suppression and a favorable prognosis in HCC patients.

**Table 1:** Correlation between PTENP1 expression and clinicopathological characteristics of HCC patients

Characteristic	Total number(N=48)	Relative PTENP1 expression		*P* value
		High (N= 24)	Low (N=24)	
Age				
<60	22	10	12	0.961
≥60	26	12	14	
Tumor size				
<3cm	31	19	12	0.035^*^
≥3cm	17	5	12	
Serum AFP (ng/ml)				
<200	37	18	19	0.852
≥200	11	6	5	
Liver cirrhosis				
Yes	32	15	17	0.54
No	16	9	7	
PVST				
Yes	4	3	1	0.296
No	44	21	23	
TNM stage				
I-II	29	18	11	0.039^*^
III-IV	19	6	13	

AFP: α-fetoprotein, PVST: portal vein system thrombosis, TNM: tumor-regional lymph node-metastasis. ^*^P < 0.05.

### PTENP1 interacts with miR-193a-3p to suppress HCC cells through the PTEN pathway

**Figure 2:**
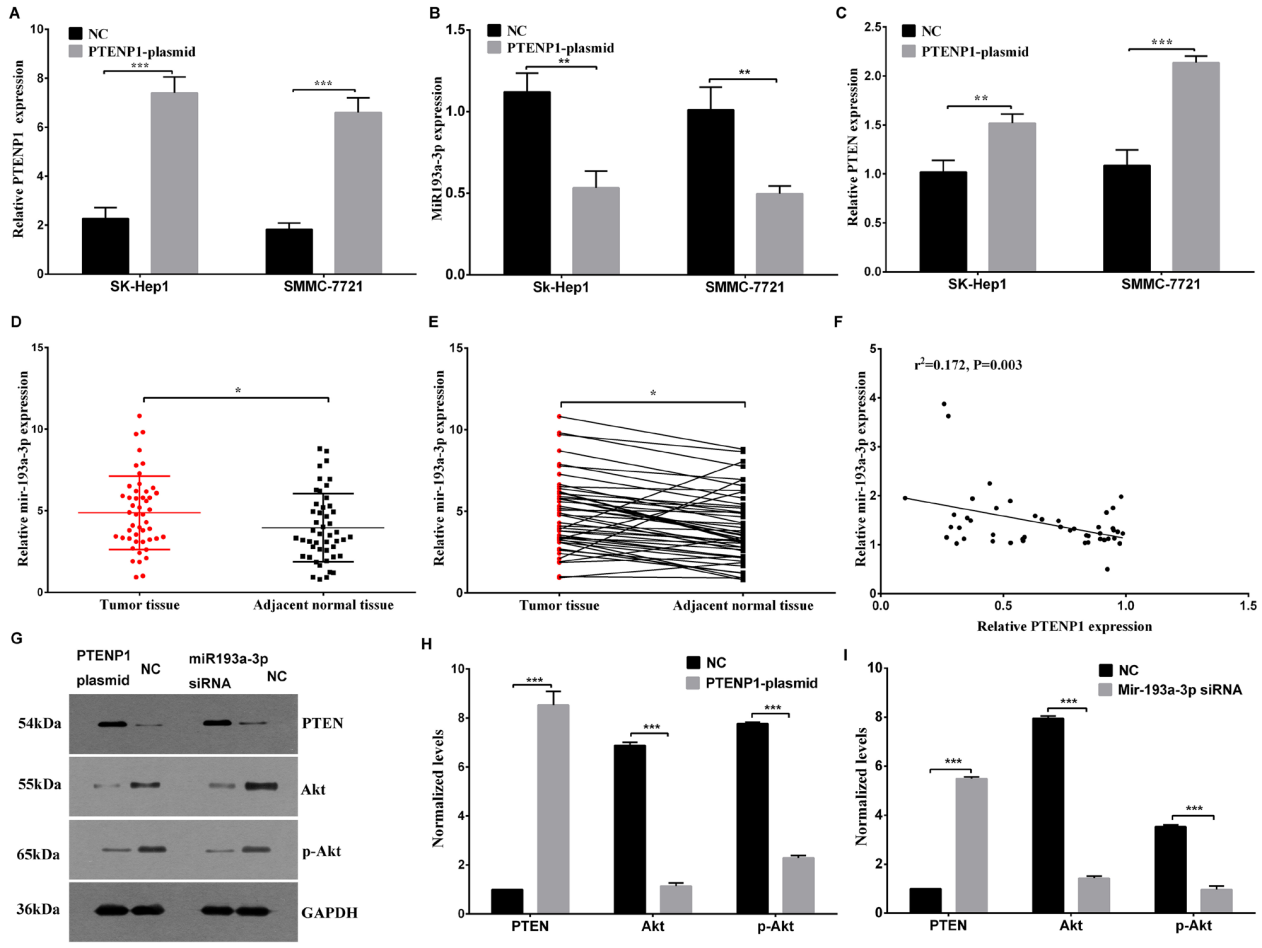
PTENP1 modulated the expression of miR-193a-3p and induced the regulation of the PTEN/Akt pathway **(A)** The PTENP1 level was analyzed after SK-Hep1 and SMMC-7721 cells were transfected with the PTENP1 plasmid or negative control plasmid. **(B)** The overexpression of PTENP1 might downregulate the miR-193a-3p expression level in SK-Hep1 and SMMC-7721 cells through ceRNA regulation mechanism. **(C)** The relative PTEN mRNA expression was evaluated in SK-Hep1 and SMMC-7721 cells transfected with the PTENP1 plasmid or negative control plasmid. **(D)** and **(E)** The miR-193a-3p expression level was higher in HCC tissues than in the paired adjacent normal tissues as determined by qPCR. **(F)** The negative association between PTENP1 and miR-193a-3p was detected by bivariate correlation analysis of 48 pairs of HCC tissues. **(G)**, **(H)** and **(I)** The PTEN/Akt pathway was analyzed by western blot assay after the upregulation of PTENP1 or the downregulation of miR-193a-3p in SMMC-7721 cells. The relative protein expression is presented. All values are the means ± SD, n=3.

The online bioinformatics prediction of FindTar3 showed putative binding sequences between PTENP1 and miR-193a-3p (Figure 4A). Therefore, we conducted experiments to investigate the expression of PTENP1 and miR-193a-3p and to confirm their target regulation both *in vitro* and *in vivo*. The expression level of PTENP1 was enhanced by transfection with the PTENP1-plasmid, which was significantly increased compared with that of the negative control in both SK-Hep1 and SMMC-7721 cells (Figure 2A). Among the 48 paired patient tissues, a higher expression level of miR-193a-3p was confirmed in the tumor tissues than that in the adjacent, nontumorous samples (Figure 2D and 2E). Interestingly, the expression level of PTENP1 was negatively correlated with the miR-193a-3p expression level among the 48 pairs of HCC tissues (Figure 2F). Next, the PTENP1-plasmid was transfected into SK-Hep1 and SMMC-7721 cells to determine the influence of PTENP1 on miR-193a-3p expression level. The result suggested that PTENP1 might affect the expression level of miR-193a-3p, and the expression level of miR-193a-3p was significantly lower in the PTENP1-plasmid group than in the NC group in HCC cells (Figure 2B).

**Figure 3:**
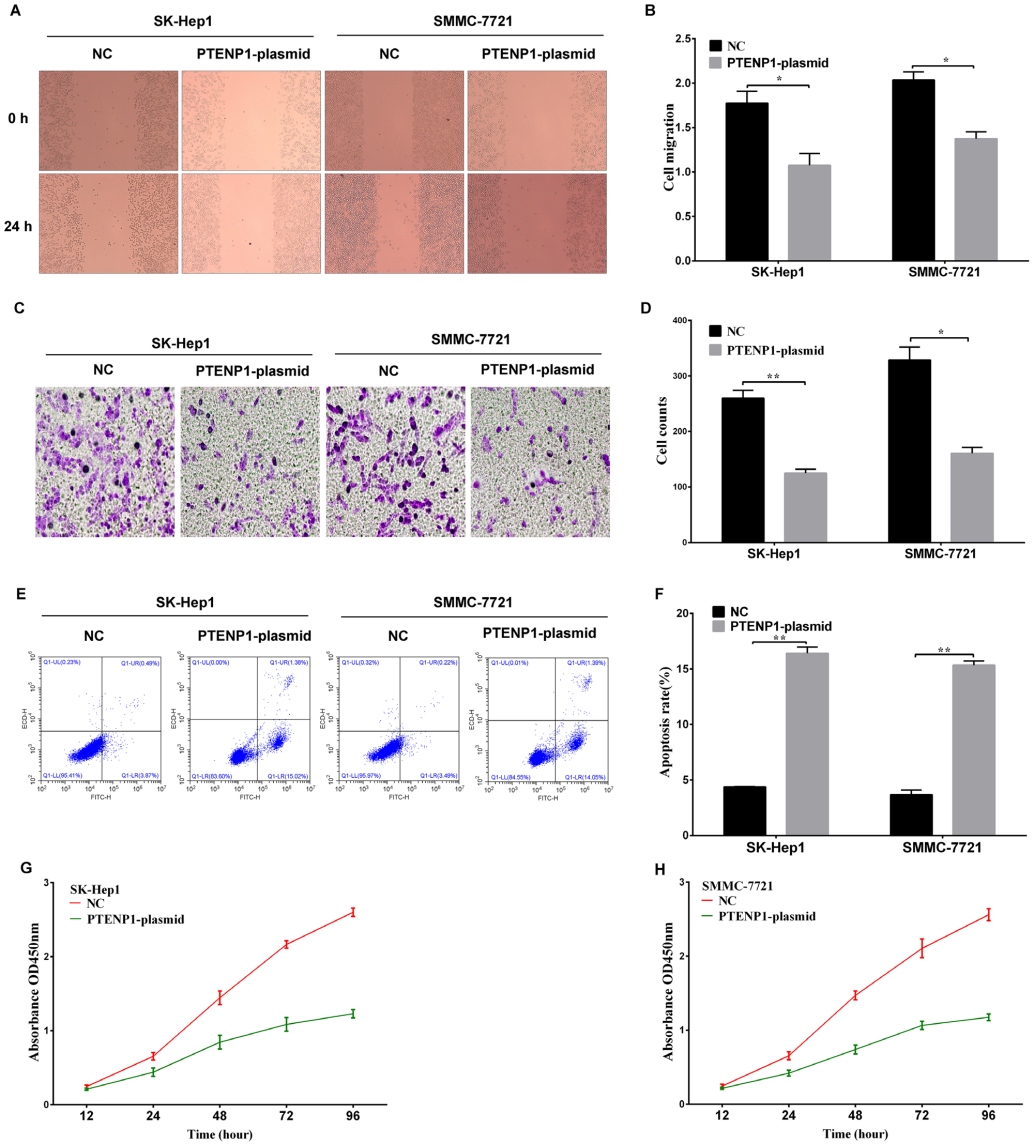
The functional analysis of PTENP1 in HCC cells **(A)** and **(B)** The wound-healing assay was applied to analyze the cell migration ability after PTENP1 was upregulated in SK-Hep1 and SMMC-7721 cells. **(C)** and **(D)** Cell invasion capacity was detected by transwell assay in SK-Hep1 and SMMC-7721 cells transfected with PTENP1-plasimd or negative control. **(E)** and **(F)** Flow cytometry assays were performed to analyze the cell apoptosis of SK-Hep1 and SMMC-7721 cells after treatment with PTENP1-plasimd or negative control. **(G)** and **(H)** MTT assay was used to evaluate the proliferation ability in SK-Hep1 and SMMC-7721 cells (P < 0.05). Data was expressed as means ± SD, n=3. ^*^P < 0.05, ^**^P < 0.01.

**Figure 4:**
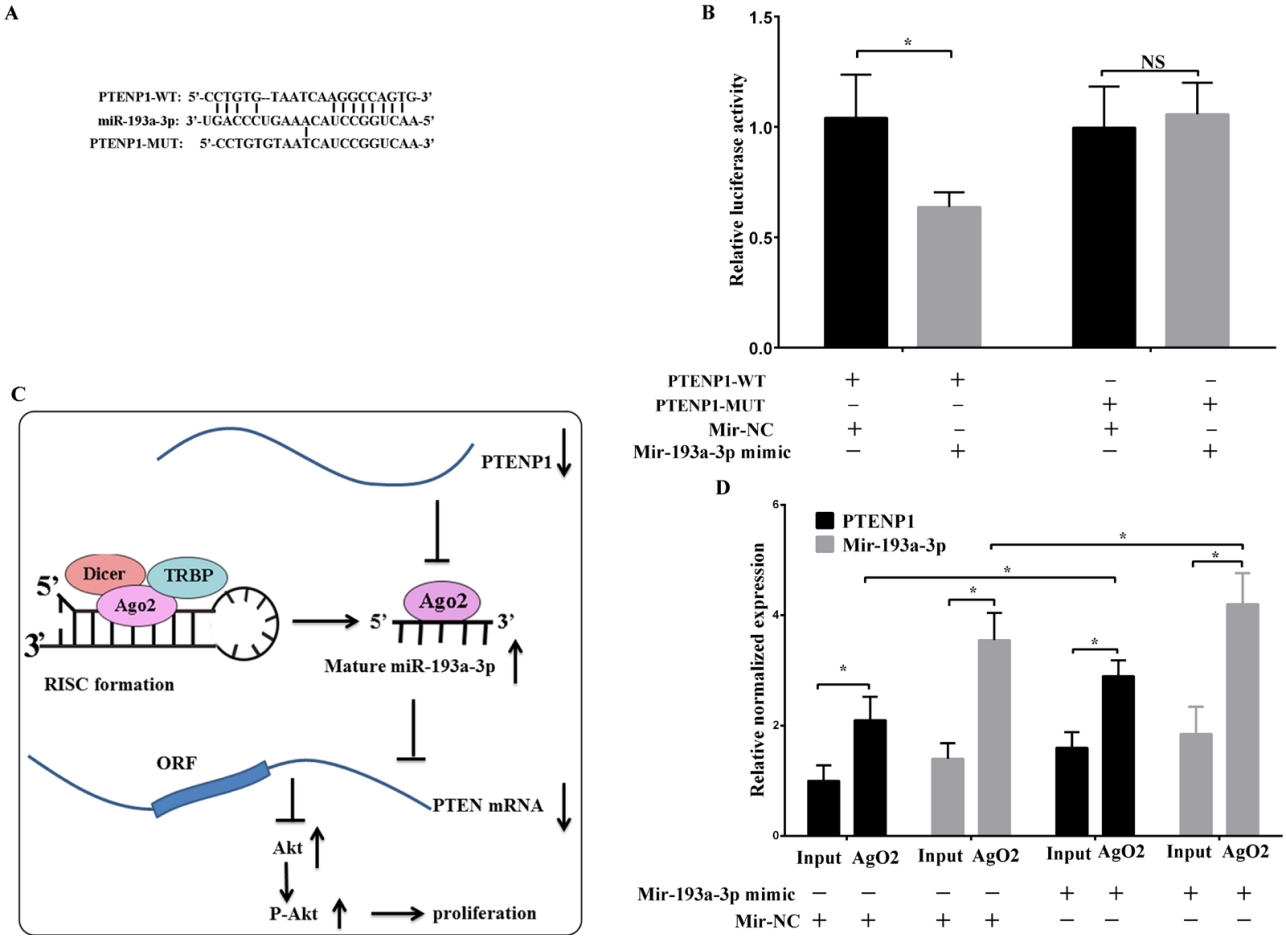
PTENP1 as a sponge directly interacted with miR-193a-3p **(A)** and **(B)** The binding sequence was predicted by FindTar3 online. The wild type and mutant PTENP1 sequences were cloned into pMir-Reporter vectors and co-transfected with miR-193a-3p mimic or miR-NC into SMMC-7721 cells. Then, the dual luciferase activity was detected. **(C)** Schematic of the proposed mechanism of PTENP1 in HCC cells. PTENP1 as a sponge interacted with miR-193a-3p and modulated the expression of PTEN/Akt pathway, leading to repress cells proliferation in HCC. **(D)** RNA immunoprecipitation with the anti-Ago2 antibody was applied to evaluate endogenous Ago2 binding to RNA. The levels of PTENP1 and miR-193a-3p were analyzed by qPCR from Ago2 precipitate. Data was expressed as means ± SD, n=3. ^*^P < 0.05, NS: no significance.

Recently, numerous studies have shown that miR-193a-3p is a tumor-promoting microRNA that promotes cell growth and migration by directly targeting the PTEN pathway [14]. Based on this previous prediction, it has been reported that PTENP1 can modulate the PTEN/Akt pathway through ceRNA regulation. The overexpression of PTENP1 elevated the relative expression level of PTEN mRNA in both HCC cells lines (Figure 2C). We also discovered that PTENP1 increased the expression level of PTEN protein and decreased the expression levels of the downstream proteins Akt and p-Akt in SMMC-7721 cells (Figure 2G and 2H). In addition, higher protein expression levels of PTEN were detected in SMMC-7721 cells transfected with miR-193b-3p siRNA than in the negative control cells (Figure 2G and 2I). The expression of miR-193a-3p was significantly lower in the cells transfected with siRNA than control by q-PCR assay (*p* < 0.01). These results indicated that PTENP1, which increased the PTEN expression level, could suppress tumorigenesis through the regulation of miR-193a-3p in HCC cells.

### PTENP1 suppressed HCC cell proliferation and induced cell apoptosis *in vitro*

Cell proliferation was investigated in SK-Hep1 and SMMC-7721 cells transfected with the PTENP1 plasmid. Cell viability was detected by the MTT assay at the absorbance value of OD450. The PTENP1-plasmid group had significantly impaired cell proliferation (*P* < 0.05; Figure 3G and 3H) during the 96-hour incubation. We detected the effect of PTENP1 on cell apoptosis by flow cytometry analysis in SK-Hep1 and SMMC-7721 cells. The result illustrated that PTENP1 markedly promoted the proportion of apoptotic cells (*P* < 0.05; Figure 3E and 3F) in HCC cells.

### PTENP1 repressed cell invasion and migration in HCC

We analyzed the effect of PTENP1 on cell invasion and migration in SK-Hep1 and SMMC-7721 cells. A low expression level of PTENP1 has been associated with tumorigenesis, cell invasion, and migration in malignant cells [17]. Transwell assays indicated that the PTENP1-plasmid-transfected group might have a lower invasion ability than the negative control group (Figure 3C and 3D). The wound-healing assay was performed to evaluate the migration capacity between the two treatment groups of SK-Hep1 and SMMC-7721 cells. These results indicated that the PTENP1-plasmid group showed a remarkably decreased migration 24-h after wounding (*P* < 0.05; Figure 3A and 3B). These results indicated that PTENP1 reduced the invasion and migration capabilities of SK-Hep1 and SMMC-7721 cells.

### PTENP1 competitively bound endogenous miR-193a-3p targeting PTEN

Based on online bioinformatics analysis (Figure 4A), we predicted that PTENP1 might bind miR-193a-3p in malignant tumors. Therefore, we performed a dual-luciferase assay to confirm the interaction between PTENP1 and miR-193a-3p. Then, we constructed two types of luciferase reporter gene vectors, the wt-PTENP1 and mut-PTENP1 (the binding sequence for miR-193a-3p was mutated) vectors, which were co-transfected into the cells with miR-193a-3p mimics or NC mimics. The results indicated that the luciferase activity of the group with the wt-PTENP1 vector co-transfected with the miR-193a-3p mimics was notably decreased compared with that of the negative control; however, there was no significant difference in the mutant group (Figure 4B).

To confirm the direct binding sequence between PTENP1 and miR-193a-3p, the RIP assay was performed in SMMC-7721 cell extracts utilizing the Ago_2_ antibody. The results demonstrated that PTENP1 and miR-193a-3p were more abundant the in RNA extracted from the Ago_2_ protein precipitate than in the IgG control (Figure 4D). Furthermore, the overexpression of miR-193a-3p caused a relative increase in the PTENP1 and miR-193a-3p expression levels in the Ago_2_ precipitate (Figure 4D). The results indicated that PTENP1 could bind directly to miR-193a-3p via the miRNA recognition site. Altogether, these results indicated that PTENP1 might act as a tumor suppressor by endogenously competing with miR-193a-3p, recovering the suppressed function of PTEN in HCC (Figure 4C).

### PTENP1 overexpression repressed HCC growth *in vivo*

**Figure 5:**
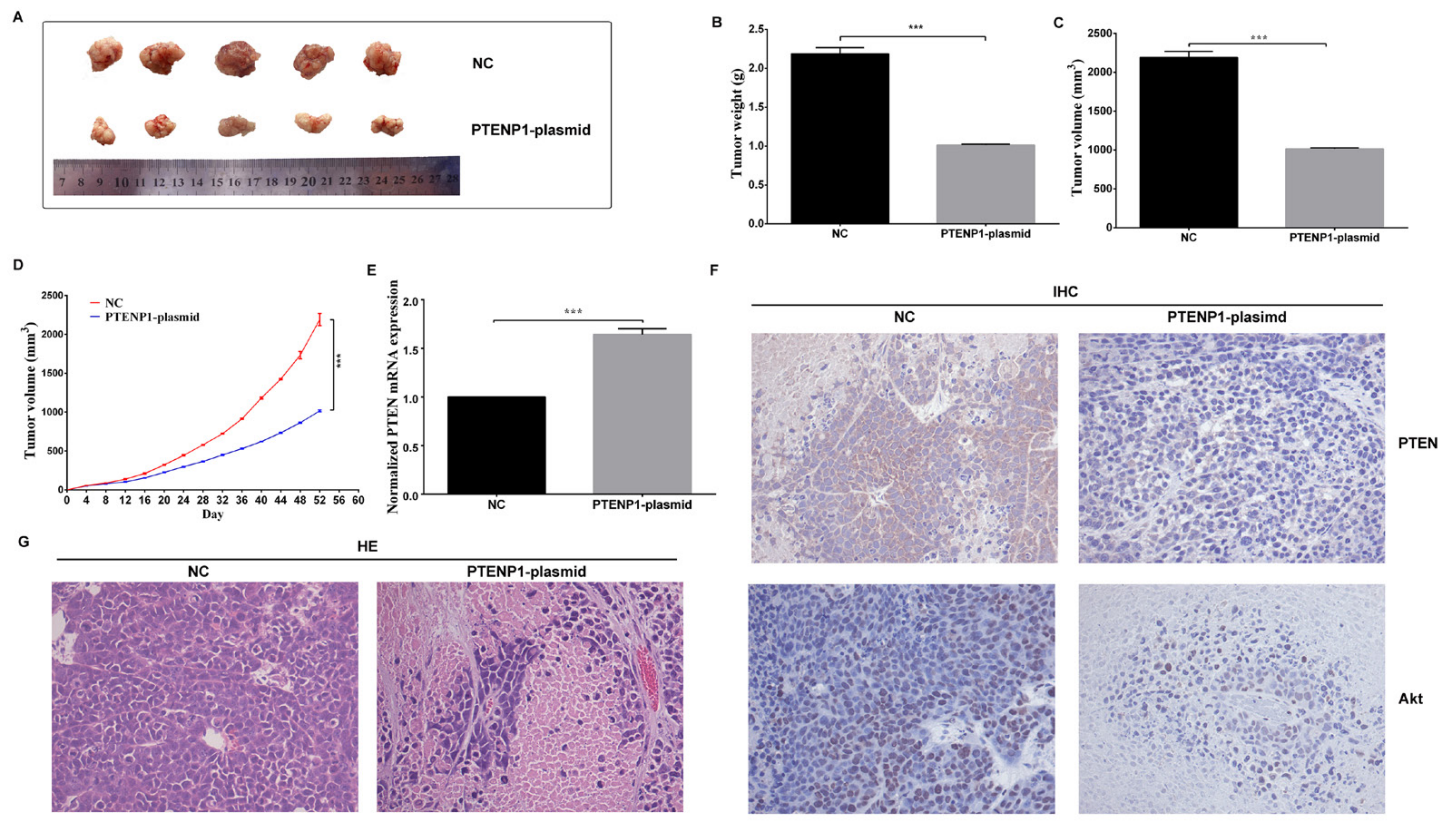
PTENP1 overexpression repressed HCC growth *in vivo* **(A)** Photographs of HCC tissues obtained from the different groups transfected with PTENP1-plasimd and negative control in nude mice. **(B)**, **(C)** and **(D)** Tumor weight, tumor volumes, and growth curve of tumor volumes were evaluated. The average volume and weight of HCC tumors in PTENP1-plasimd group were significantly lower than those of in the NC group. **(E)** *In vivo*, the overexpression of PTENP1 increased the PTEN level than the negative control. **(F)** The protein expression of PTEN, and Akt were analyzed by IHC assay in HCC. **(G)** Sections of xenograft tumors were stained by hematoxylin and eosin (HE). The PTENP1-plasimd group increased more necrosis cells than the control. Data was expressed as means ± SD, n=3. ^***^P < 0.001.

To investigate the important roles of PTENP1 on tumorigenicity *in vivo*, a xenograft tumor model was constructed by injecting transfected cells into mice. Based on the periodic observation of tumor growth, tumor volumes in the PTENP1-plasmid group were significantly smaller than those in the NC group (Figure 5D). Fifty-two days later, the mice were executed, and the tumors were harvested (Figure 5A). We subsequently analyzed the weight and volumes of the tumors in the PTENP1-plasmid group, which were significantly decreased compared with those in the NC group (Figure 5B and 5C). *In vivo*, the overexpression of PTENP1 substantially increased the PTEN expression level when compared to that of the NC group (Figure 5E). The protein expression levels of PTEN and Akt were analyzed by IHC staining in HCC tissues. In addition, the results demonstrated that the PTEN expression levels were higher in the PTENP1-plasmid group than those in the NC group. And Akt levels were lower than control (Figure 5F). In the hematoxylin and eosin staining results, the high expression level of PTENP1 promoted the necrosis of HCC *in vivo* (Figure 5G).

## DISCUSSION

The dysregulation of lncRNAs is related to various biological functions of tumors [18–20]. It had been reported that PTENP1 can suppress cell proliferation and promote cell apoptosis in breast cancer [10], gastric cancer [11], oral squamous cell carcinoma [21], renal cell carcinoma [17], etc. In addition, Liu L *et al*. discovered that miR-193a-3p can inhibit tumor growth by directly targeting PTEN [14]. In our recent study, we discovered a relatively lower expression level of PTENP1 and higher expression level of miR-193a-3p in HCC samples than in adjacent normal tissues. Furthermore, there was a low expression level of PTENP1 and a high expression level of miR-193a-3p in HCC tissues, which related to tumor size and TNM stage, leading to a poor prognosis. PTENP1 can upregulate PTEN via its ceRNA interaction with miR-193a-3p.

Subsequently, we detected the functional significance of PTENP1 in cells with the transfection of the PTENP1-plasmid into HCC cell lines. The data indicated that PTENP1 overexpression inhibited cell proliferation, promoted cell apoptosis, repressed cell invasion, and suppressed cell migration. In addition, in animal experiments, we also confirmed the suppression effect of PTENP1 in tumors.

LncRNAs acting as endogenous “sponges” or ceRNA interact with miRNA and exert their biological function by binding to their target [22, 23]. The lncRNA PTENP1 exerts its tumor suppressor function by inhibiting miR-193a-3p in the RISC complex. Moreover, PTENP1 has a binding site for miR-193a-3p, which plays an important role in suppressing miR-193a-3p through the RISC complex in the transcriptional regulatory pathway. To confirm that PTENP1 is an endogenous “sponge” interacting with miR-193a-3p, we conducted a variety of experiments. Our results suggest that the overexpression of PTENP1 might repress miR-193a-3p expression levels in HCC cells. In addition, the luciferase reporter assay demonstrated that PTENP1 can directly downregulate miR-193a-3p levels. Subsequently, an RNA immunoprecipitation (RIP) assay was performed to detect the endogenous interaction between PTENP1 and miR-193a-3p.

Finally, PTENP1 modulated the miR-193a-3p expression level, affecting the regulation of the downstream PTEN/Akt pathway. PTEN expression levels were downregulated in tumor tissues, which was related to tumor suppression [24]. All the results show that PTENP1 acts as an endogenous sponge, mediating the expression level of its target miR-193a-3p, which regulates the downstream PTEN expression level.

## MATERIALS AND METHODS

### Patients and tissue samples

The 48 paired samples of HCC tissue and adjacent normal tissue came from patients receiving surgical resection at the Wuhan University of Zhongnan Hospital. And this study was approved by the Ethics Committee of Zhongnan Hospital. For both the human and animal study, all methods were performed in accordance with the relevant ethical requirements. All patients signed informed consents, and were followed by a 36-month survival investigation, including overall survival (OS) and recurrence free survival (RFS). The basic characteristics of the patients are shown in Table 1.

### Cell culture

The Sk-Hep-1 and SMMC-7721 cells were cultivated from the Cell Bank of Type Culture Collection (CBTCC, Chinese Academy of Sciences, Shanghai, China). The cells were incubated and maintained in RPMI 1640 medium (Gibco, Darmstadt, Germany) supplemented with 10% fetal bovine serum in a humidified incubator of 5% CO_2_ at 37 °C.

### Western blots

Total cell lysates were prepared with RIPA Lysate Buffer (Thermo Fisher Scientific, Rockford, USA). The proteins were separated by SDS-PAGE (4% stacking and 10% separating gels) and then transferred to PVDF membranes (Millipore, Billerica, USA). TBST with 5% skim milk powder was used for blocking the PVDF membranes. Then, the blots were incubated with primary antibodies (Abcam, London, UK) overnight at 4 °C. After incubating with secondary antibodies, the proteins were visualized by enhanced chemiluminescence. The relative protein levels were quantified by ImageJ software.

### Quantitative real-time PCR

Total RNA was isolated from tissues and HCC cell lines by TRIzol reagent (Invitrogen, CA, USA). RNA was reverse transcribed to cDNA using Reverse Transcription Kit (Invitrogen, CA, USA). Then, real-time PCR analysis was conducted with SYBR-Green (Invitrogen, CA, USA). The relative expression of the RNA was evaluated by using the 2^-ΔΔCt^ method (normalized to the internal control). The specific primers are shown in Table 2.

**Table 2:** PCR amplification primer sequences

Target	Primer	Sequence
PTENP1	F primer	5’ - TCAGAACATGGCATACACCAA-3’
	R primer	5’ - TGATGACGTCCGATTTTTCA-3’
miR-193a-3p	F primer	5’ - GCATAACTGGCCTACAAAGT-3’
	R primer	5’ - GTGCAGGGTCCGAGGT-3’
U6	F primer	5’ - CTCGCTTCGGCAGCACA-3’
	R primer	5’ - AACGCTTCACGAATTTGCGT-3’
PTEN	F primer	5’ - CCAAGCTTATGACAGCCATCATC-3’
	R primer	5’ - CGCGGATCCTCAGACTTTTGTAA-3’
GAPDH	F primer	5’ - GGAGCGAGATCCCTCCAAAAT-3’
	R primer	5’ - GGCTGTTGTCATACTTCTCATGG-3’

PCR: polymerase chain reaction, RT: real time, F: forward, R: reverse.

### Cell transfection assay and RNA interference

The PTENP1 plasmid was constructed by inserting PTENP1 (subcloning sequence) into a pCDNA3.1 vector (Invitrogen, CA, USA). Then, the plasmid was transfected into the cells by using Lipofectamine 2000 reagent (Invitrogen, CA, USA) according to the manufacturer's instructions. The miR-193a-3p siRNA (Sigma, St. Louis, MO, USA) was used to knockdown the miR-193a-3p expression levels. Then, the miR-193a-3p siRNA vector was transfected into cells by Lipofectamine 2000 for incubation. After a 24-h transfection period, cells were harvested for study.

### MTT assays

Cell proliferation ability was investigated by the 3-(4,5-dimethylthiazol-2-yl)-2,5-diphenyl-tetrazolium bromide (MTT, Boster, Wuhan, China) reagent. The cells were seeded at 3 × 10^4^ cells/well in 96-well plates, and then, 20 μL of MTT (2.5 mg/ml) was added to each well. We used 200 ml of DMSO to dissolve the formazan after incubation in 5% CO2 at 37 °C for 12, 24, 48, 72 and 96 h. Subsequently, the absorbance at 450 nm was measured by a microplate reader (Thermo Fisher Scientific, Waltham, MA, USA).

### Cell invasion assay

Cell invasion was analyzed by transwell chambers (Corning, NY, USA) with 8-μm pore filters. The SK-Hep1 and SMMC-7721 cells in serum-free medium were cultured in the upper chambers that were precoated with Matrigel (Becton, Dickinson and Company, NJ, USA). DMEM containing 10% FBS was added to the lower chambers. After incubation at 37 °C for 24 h, the non-invading cells that remained on the upper membrane were removed. Invading cells were fixed with 4% paraformaldehyde, stained with 0.1% crystal violet, and counted by using a phase-contrast microscope.

### Cell migration assay

Cells (1×10^6^ cells/well) were cultured in six-well plates. The monolayered cells were separated with standardized wound scratching by a sterile pipette tip. After the cells were incubated in serum-free medium for 24 h, the migrating distance was measured and photographed by phase-contrast microscopy (×4).

### Apoptosis assay

Flow cytometry was used to analyze cell apoptosis by staining with Annexin V-FITC and PI (Annexin V-FITC Apoptosis Detection Kit, Sigma, St. Louis, MO, USA). Then, the stained cells were detected by a flow cytometer, and the results were analyzed by using FlowJo V10 software (BD Bioscience, San Jose, CA, USA).

### Luciferase reporter assay

Sk-Hep-1 and SMMC-7721 cells were cultured overnight. Then, cells were co-transfected with the pMir-Reporter-PTENP1-WT, the pMir-Reporter-PTENP1-MUT and miR-193a-3p mimic, or the negative control plasmid. The reporter plasmid was transfected into cells by Lipofectamine 2000 (Invitrogen Co., Carlsbad, CA, USA) for 48 h. Then, luciferase activity was detected by the dual-luciferase reporter gene assay system (Promega, Madison, WI, USA).

### RNA immunoprecipitation (RIP)

Sk-Hep-1 and SMMC-7721 cell lysates were used for RIP with an Imprint RNA Immunoprecipitation Kit (Sigma, St. Louis, MO, USA). RIP buffer was added to magnetic beads conjugated to either a human anti-Ago2 antibody (Sigma, St. Louis, MO, USA) or negative control IgG and was used to precipitate the cell extracts. The expression levels of PTENP1 and miR-193a-3p in the precipitates were analyzed by qPCR.

### Animal experiments

Ten male immune-deficient nude mice (BALB/c-nu), approximately 4 weeks old, were purchased from the animal laboratory (Vantong Lihua Experimental Company, Beijing, China) and allowed free access to water and food. All mice were randomly assigned to either the NC group or the PTENP1-plasmid group, which was approved by the Ethics Committee of Wuhan University of Zhongnan Hospital. Mice were injected in the subcutaneous axilla with 0.2 ml of SMMC-7721 cells (NC or PTENP1-plasmid transfection) at a concentration of 1 × 10^6^ cells/ml diluted in PBS [15]. Then, tumor volume was measured by calipers every four days and calculated by the following formula: volume = (length × width^2^)/2 [16].

### Immunohistochemical (IHC) and hematoxylin and eosin (HE) analysis of specimens

All the tumor tissues were fixed in 4% formalin and then embedded in 4-μm-thick paraffin sections. Then, the paraffin sections were incubated with PTEN or Akt primary antibodies overnight at 4 °C. 3,3-Diaminobenzidine solution and hematoxylin (Aspen, Wuhan, China) were used to stain the sections. A Nikon Eclipse 80i microscope (Nikon, Tokyo, Japan) was used to take photographs. Sections were stained with 3,3-diaminobenzidine solution for 3 min, and the nuclei were counterstained with hematoxylin. The paraffin embedded tumor sections were stained with hematoxylin and eosin. Subsequently, the results were visualized with a microscope.

### Statistical analyses

All data are presented as the means ± SD, which were collected from three independent experiments. The SPSS 21.0 software (IBM, Chicago, IL, USA) was used for statistical analyses. The differences or correlations between groups, such as for cell viability, cell apoptosis, cell proliferation and cell invasion, were evaluated by using one-way analysis of variance (ANOVA) or independent sample t-tests. The expression levels of PTENP1 and miR-193a-3p in the tumor tissues and adjacent normal tissues were compared by the paired sample t-test. The clinical data from patients were estimated by the chi-square test. OS and RFS were statistically analyzed by the Kaplan-Meier test. A value of *P* < 0.05 was regarded as statistically significant.

## CONCLUSION

In summary, PTENP1, a tumor suppressor, was low in HCC tissues and has the potential to be a prognostic biomarker of HCC. Moreover, PTENP1 was discovered to act as a sponge for miR-193a-3p, regulating the downstream PTEN/Akt pathway. PTENP1 mediated cell proliferation, migration and invasion by endogenously competing with miR-193a-3p through PTEN in HCC. The results suggest that PTENP1 may provide a new prospect for diagnostics and drug targets in HCC.
